# Comparison of Routes of Administration, Frequency, and Duration of Favipiravir Treatment in Mouse and Guinea Pig Models of Ebola Virus Disease

**DOI:** 10.3390/v16071101

**Published:** 2024-07-09

**Authors:** Dylan M. Johnson, Terry Juelich, Lihong Zhang, Jennifer K. Smith, Birte K. Kalveram, David Perez, Jeanon Smith, Michael R. Grimes, Tania Garron, Maricela Torres, Shane Massey, Trevor Brasel, David W. C. Beasley, Alex N. Freiberg, Jason E. Comer

**Affiliations:** 1 Department of Microbiology and Immunology, University of Texas Medical Branch at Galveston, Galveston, TX 77555, USA; 2 Department of Biotechnology & Bioengineering, Sandia National Laboratories, Livermore, CA 945501, USA; 3 Department of Pathology, University of Texas Medical Branch at Galveston, Galveston, TX 77555, USA; tljuelic@utmb.edu (T.J.);; 4 Office of Biosafety, Texas A&M University, College Station, TX 77843, USA; 5 Office of Regulated Nonclinical Studies, University of Texas Medical Branch at Galveston, Galveston, TX 77555, USAchmassey@utmb.edu (S.M.); 6 Center for Molecular and Translational Human Infectious Diseases Research, Houston Methodist, Houston, TX 77030, USA; mrgrimes@houston-methodist.org

**Keywords:** filovirus, Ebola virus, favipiravir, T-705, EBOV, animal model

## Abstract

Favipiravir is a ribonucleoside analogue that has been explored as a therapeutic for the treatment of Ebola Virus Disease (EVD). Promising data from rodent models has informed nonhuman primate trials, as well as evaluation in patients during the 2013–2016 West African EVD outbreak of favipiravir treatment. However, mixed results from these studies hindered regulatory approval of favipiravir for the indication of EVD. This study examined the influence of route of administration, duration of treatment, and treatment schedule of favipiravir in immune competent mouse and guinea pig models using rodent-adapted *Zaire ebolavirus* (EBOV). A dose of 300 mg/kg/day of favipiravir with an 8-day treatment was found to be fully effective at preventing lethal EVD-like disease in BALB/c mice regardless of route of administration (oral, intraperitoneal, or subcutaneous) or whether it was provided as a once-daily dose or a twice-daily split dose. Preclinical data generated in guinea pigs demonstrates that an 8-day treatment of 300 mg/kg/day of favipiravir reduces mortality following EBOV challenge regardless of route of treatment or duration of treatments for 8, 11, or 15 days. This work supports the future translational development of favipiravir as an EVD therapeutic.

## 1. Introduction

### 1.1. Zaire ebolavirus and Sudan ebolavirus Epidemiology and Treatments

Viruses in the *Filoviridae* family, including *Zaire ebolavirus* (EBOV), are emerging viruses that have recently caused several outbreaks with high morbidity and mortality. EBOV first emerged in Zaire in 1976 and has caused sporadic outbreaks since [1,2]. In 2013–2016, a West African outbreak of EBOV resulted in 28,610 reported cases with an overall 39% case-fatality rate [2,3].

Recently, there have been several significant advances in vaccines and therapeutics for the prevention and treatment of Ebola Virus Disease (EVD). The World Health Organization (WHO) issued treatment guidelines for EVD that call for antibody treatment with mAb114 (Ansuvimab, EBANGA™) or REGN-EB3 (atoltivimab/maftivimab/odesivimab-ebgn, INMAZEB^®^) [4,5]. Additionally, the United States (US) Food and Drug Administration (FDA) and the European Medicines Agency (EMA) granted approval for mAb114, REGN-EB3, and Ervebo, a VSV-vectored live attenuated vaccine against EBOV, as an EVD prophylactic measure [6,7,8]. Additionally, the EMA has approved Zabdeno/Mvabea, a replication incompetent two-dose EVD vaccine [9]. However, while mAb114 and REGN-EB3 reduce the risk of death from EVD, there is still significant morbidity (primarily fever, diarrhea, abdominal pain, headache, vomiting, conjunctival injection, hemorrhage, and cough) and mortality (35.1% and 33.5%, respectively, in clinical trials) among treated patients [10,11]. Both mAb114 and REGN-EB3 are monoclonal antibody drugs which have several downsides: they are costly to manufacture, have limited shelf-life, and require cold-chain storage. Monoclonal antibody drugs target viral (glyco-)proteins and are therefore highly specific, usually with their usage limited to one viral species. Both mAb114 and REGN-EB3 are only indicated for the treatment of EBOV [5]. MBP134, a monoclonal antibody cocktail with experimental efficacy against SUDV, has recently been identified as a candidate for advancement to clinical trials [12].

### 1.2. Favipiravir as an Antiviral Medication

Favipiravir (T-705, Avigan^®^) is a pyrazineccarboxamide derivative which is a prodrug that is metabolized to favipiravir-ribofuranosyl-5′-triphosphate and has been proposed as a broad-spectrum antiviral ribonucleoside analogue [13]. Favipiravir is approved in Japan for the treatment of emerging Influenza virus strains that are resistant to other commonly used antiviral drugs [14]. The exact mechanism of action (MOA) for favipiravir is likely by hindering the RNA-dependent RNA-polymerase of filoviruses; however, the exact MOA is unknown. There is evidence from non-human primate studies suggesting that, at sub-therapeutic doses, RNA chain termination and possibly error catastrophe occur [13,15,16,17]. Favipiravir has been investigated for the treatment of EVD and for the resolution of post-acute EBOV persistence in humans and has shown acceptable tolerability, but these studies do not provide statistically significant evidence for treatment efficacy, perhaps as a result of the serum concentrations achieved [18,19,20,21].

### 1.3. Favipiravir for the Treatment of Filoviral Diseases

The in vivo antiviral efficacy of favipiravir has been evaluated in multiple rodent and non-human primate models for filovirus infection (Table 1) [16,17,22,23,24,25]. Favipiravir treatment prevented lethal SUDV disease in a STAT-1 deficient mouse model when treatment was initiated immediately following infection and 300 mg/kg/day was administered orally as a split dose twice daily [26]. Favipiravir treatment of SUDV infection in Hartley guinea pigs resulted in survival of 38% of animals when 150 mg/kg/day was given for 7 days starting 2 days post challenge, and survival of 83–100% when a 7-day treatment with a dose of 300 mg/kg/day was initiated between 1 and 5 days following challenge, compared to no survival in the control group [23]. In a model of EVD in interferon alpha/beta receptor deficient mice on an A129 background, following aerosol infection, treatment with 300 mg/kg/day of favipiravir was administered orally as a split dose twice daily for 14 days and provided complete protection against lethal challenge [22]. Another trial of favipiravir treatment of an intranasal model of EVD in interferon alpha/beta receptor-deficient mice on a C57BL/6 background demonstrated complete protection against lethal disease when 300 mg/kg/day was used to treat from days 6 to 13, while no protection was achieved when treatment was delated until day 8 [24]. Treatment with favipiravir reduced mortality of intraperitoneally administered EBOV in an interferon alpha/beta and gamma receptor-deficient mouse model when treatment was started following infection and 300 mg/kg/day was administered orally as a split dose twice daily [27]. For Marburg virus (MARV), 300 mg/kg/day of favipiravir administered for 8 days provided complete protection when started within two days of challenge, and partial protection up to four days post challenge in a BALB/c mouse model [16]. Similarly, non-human primates (NHPs) treated with a 250 mg/kg loading dose on the day of MARV challenge, followed by a 150 mg/kg twice daily dose of favipiravir, had survival of 83% (compared to no survival in the untreated group) [28].

The results from non-human primates for favipiravir treatment of EVD-like disease are less promising. Cynomolgus macaques treated with a 400 mg/kg loading dose 3 days prior to challenge followed by 200 mg/kg/day for 13 days had an increased mean time-to-death compared to non-protected animals, and 1 of 6 favipiravir-treated animals survived challenge [28]. Conversely, loading doses of 125 mg/kg and 250 mg/kg on the day of challenge followed by twice-daily treatment with 150 mg/kg/day or 75 mg/kg/day for 13 days did not improve survival but displayed a moderate dose-dependent increase in mean time-to-death [28]. Another study of cynomolgus macaques demonstrated treatment with a loading dose followed by 13 days of maintenance doses of 200/100 mg/kg/12 h, 250/150 mg/kg/12 h, and 250/180 mg/kg/12 h starting 2 days prior to challenge showed no effect for the low dose and 40% and 60% survival in the mid- and high-dose groups, respectively [17,25,29]. Overall, the fact that favipiravir is well tolerated in humans, limited efficacy data from human and non-human primate studies, and efficacy data from rodent studies justifies further development of this compound as a therapeutic for the treatment of EVD.

### 1.4. Rationale

A mouse model of EVD based on a challenge of BALB/c mice with mouse-adapted EBOV (maEBOV) and a guinea pig model with guinea pig-adapted EBOV (gpaEBOV) have been described as outlined above [30,31]. This study investigated the optimal dose, schedule of dosing, and dosing route for favipiravir in these rodent models. In this manuscript, we describe the pre-clinical testing of favipiravir against maEBOV in BALB/c immune-competent mice and against gpaEBOV in Hartley outbred guinea pigs. Dosing routes (including subcutaneous [SC], intraperitoneal [IP], and oral [PO]), dosing schedules (once a day [QD] or twice a day [BID]), and different durations of treatments are compared. Regulatory approval can be a barrier to manufacturing and stockpiling of EVD therapeutics. Both the FDA and EMA have approval routes for alternative licensing of therapeutics when human efficacy trials cannot be performed. Typically, these require efficacy in animal models following Good Laboratory Practice (GLP)-like standards for data collection and study documentation. Data generated on the guinea pig model of EVD in this study was produced with GLP-like standards in support of the development of translational guinea pig EVD models and the inclusion of favipiravir in future regulated studies. A dose of 300 mg/kg was used to build upon the successes of previous studies of rodent filoviral infection treatment with favipiravir [16,24,26,27,32]. Based on the findings that favipiravir treatment should be initiated as soon as possible following infection [33], treatment was started at 1 h post challenge to mimic treatment following a known exposure event in a nosocomial or laboratory setting.

## 2. Materials and Methods

### 2.1. Ethical Approval

All animal experiments were approved by the Institutional Animal Care and Use Committee of The University of Texas Medical Branch (protocol number 1407044 and 1712071) and performed following the guidelines of the Association for Assessment and Accreditation of Laboratory Animal Care International (AAALAC) by certified staff in an AAALAC-approved facility. All experimental work was performed in the Robert E. Shope and Galveston National Laboratory animal biosafety level 4 (ABSL4) laboratories at the University of Texas Medical Branch following protocols approved by the Institutional Biosafety Committee.

### 2.2. Virus Characterization and Animal Models

**Figure 1:**
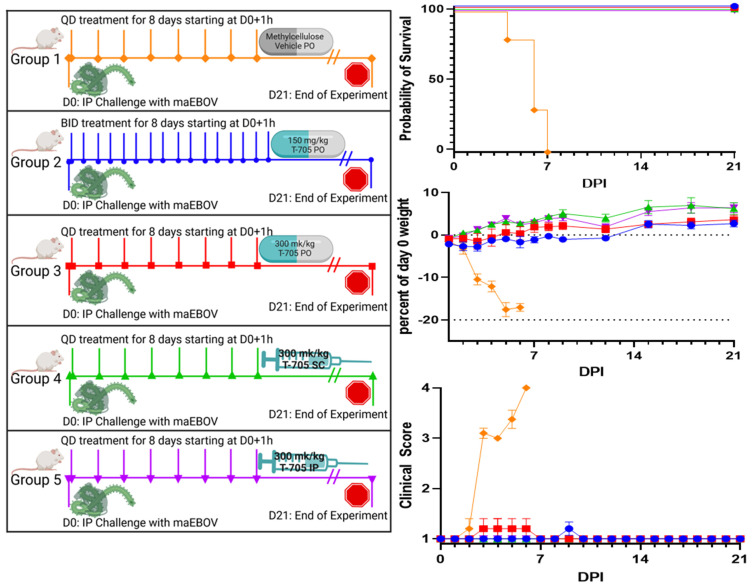
Groups (n = 10, 5 males, 5 females) of BALB/c mice were challenged with a 2.9 × 10^2^ PFU dose of 1000 PFU of maEBOV at day 0 (D0) (left panel schematic). One hour post infection, treatment with 300 mg/kg of favipiravir daily given either QD (red squares, green triangles, and purple inverted triangles) or split into BID doses of 150 mg/kg (blue circles) was initiated. A control group was sham treated with vehicle only (orange diamonds). Treatment was delivered PO (orange diamonds, blue circles, red squares), SC (green triangles), or IP (purple inverted triangles). Animals were monitored for 21 days for morbidity and mortality (top right panel; *p* < 0.0001 by Mantel–Cox log-rank test), percent change in weight from D0 (right middle panel), and clinical score (right bottom panel).

**Figure 2:**
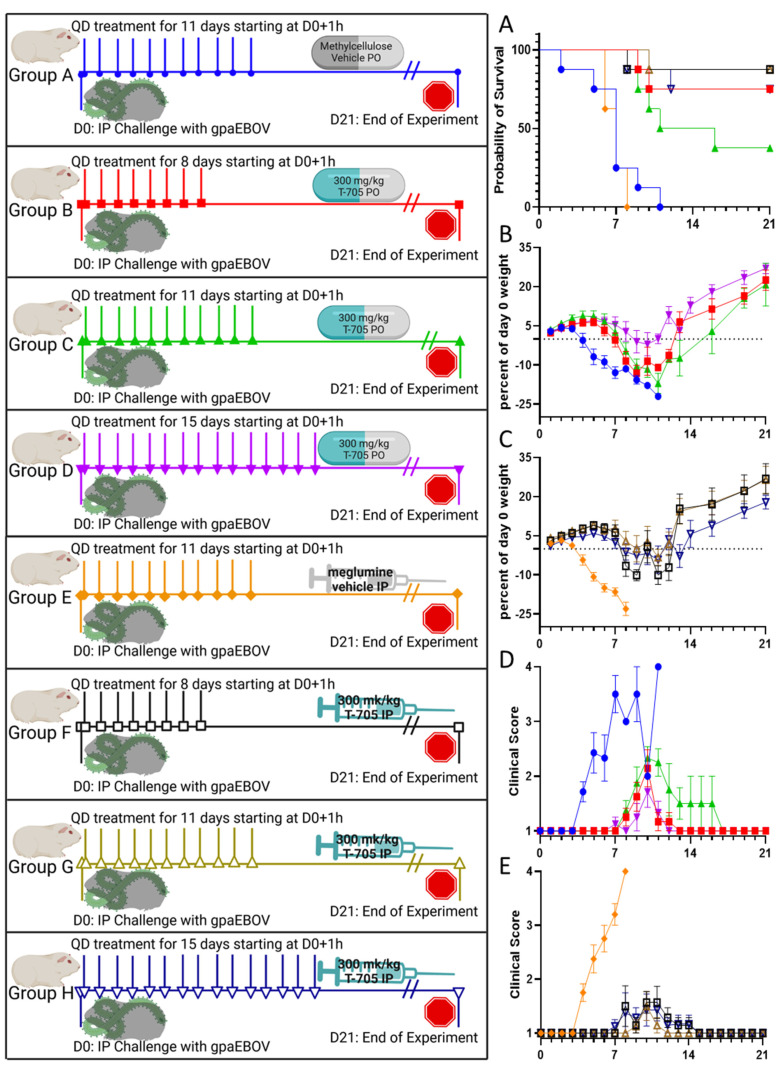

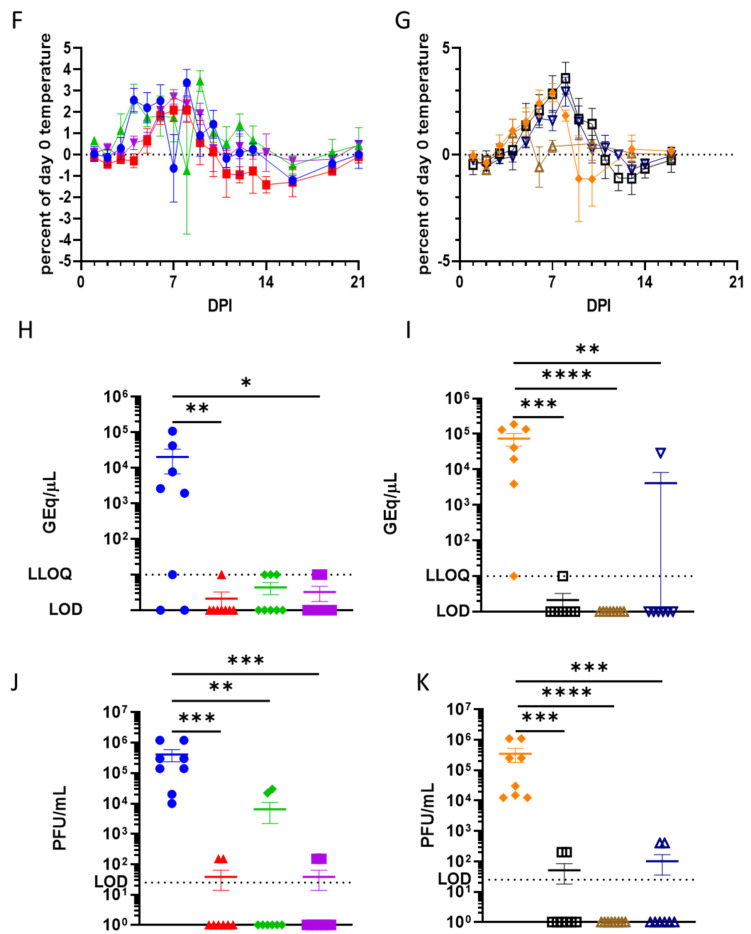
Groups (n = 8, 4 males, 4 females) of Hartley outbred guinea pigs were challenged with a target dose of 1000 PFU of gpaEBOV at D0 (left panel schematic). After an hour, they were treated QD with 300 mg/kg of favipiravir delivered PO (red squares, green triangles, purple inverted triangles) or IP (black open squares, brown open triangles, navy open inverted triangles) for 8 days (red squares, black open squares), 11 days (green triangles, brown open triangles), or 15 days (purple inverted triangles, navy open inverted triangles). A control group was sham treated PO (blue circles) or IP (orange diamonds) with vehicle only QD for 11 days. Animals were followed for 21 days for survival (panel **A**; *p* < 0.0001 by Mantel–Cox log-rank test), percent change in weight from D0 (PO treatment in panel (**B**); IP treatment in panel (**C**)), (clinical score (PO treatment in panel (**D**); IP treatment in panel (**E**)). Temperatures were taken by IPTT-300 RFID transponder for guinea pigs treated PO (panel (**F**)) or IP (panel (**G**)). Blood was collected at the time of euthanasia and serum was either used for RNA extraction and qRT-PCR viral load quantification (PO panel (**H**), IP panel (**I**)) or stored frozen at −80 °C for plaque assay quantification (PO panel (**J**), IP panel (**K**). Differences in qRT-PCR GEq/μL and PFU/mL were analyzed by Kruskal–Wallis test with Dunnet’s post hoc comparison to vehicle-treated controls. * *p* < 0.05, ** *p* < 0.01, *** *p* < 0.001, **** *p* < 0.0001.

Six- to eight-week-old BALB/c mice were purchased from Jackson laboratories (Bar Harbor, ME). Hartley guinea pigs (250–300 g) were purchased from Charles River Laboratories (Wilmington, MA). All animals were group housed and provided food and water ad libitum. Animals were given a minimum of 72 h to acclimate to ABSL4 housing prior to challenge. All procedures, including challenge and treatments, were performed under isoflurane anesthesia. The challenge virus was administered via the intraperitoneal (IP) route. Animals (n = 10 for mice and n = 8 for guinea pigs; equal sex) were observed at least once daily for clinical score and weighed at the times indicated in Figure 1 and Figure 2. Clinical score was measured on a 4-point scale, with “1” indicating normal health, “2” indicating lethargy or decreased grooming, “3” indicating the addition of hunched posture or orbital tightening along with lethargy or decreased grooming, and “4” meeting humane euthanasia criteria due to paralysis, reluctance to move when stimulated, inability to access feed or water, or loss of 20% of body weight. BMDS IPTT-300 programable radio frequency identification transponders (RFIDs) (Avidity Science, Waterford, WI) were used to monitor body temperature of guinea pigs at the times indicated in Figure 2.

Challenge studies with BALB/c mice were completed with a target dose of 1000 plaque forming units (PFU) of mouse-adapted *Zaire ebolavirus* (maEBOV). Hartley outbred guinea pigs were challenged with a target dose of 1000 PFU of guinea pig-adapted *Zaire ebolavirus* (gpaEBOV).

### 2.3. Favipiravir Dosing

Favipiravir was obtained from Adooq Bioscience (Irvine, CA, USA) and formulated in 0.5% methylcellulose in sterile water for PO dosing or with 74.6 mg/mL meglumine in sterile water for subcutaneous (SC) or IP dosing as previously described [34]. Animals were dosed based on their average body weight at the start of the study as indicated in the corresponding figures. Based on previous studies investigating the effects of favipiravir against filoviruses and arenaviruses, 150 mg/kg and 300 mg/kg doses were used [23,32,34,35].

### 2.4. Viral Quantification by qRT-PCR and Plaque Assay

Plaque assay was conducted as previously described [27]. Briefly, monolayers of Vero E6 cells (ATCC CRL-1586, Manassas, VA, USA) were infected with dilutions of serum for 1 h before being overlaid with 0.5% agarose and incubated for 10 days at 37 °C, 5% CO_2_. A neutral red solution (Sigma-Aldrich, St. Louis, MO, USA) was added and the plates were incubated for an additional day before counting plaques. The limit of detection (LOD) was 25 PFU/mL of serum.

Serum was collected and inactivated with TRIzol LS (Invitrogen, Waltham, MA, USA). RNA was isolated using a Direct-zol kit according to the manufacturer’s direction (Zymo Research, Irvine, CA, USA). qRT-PCR on RNA purified from serum was performed with a QuantiFast Probe RT-PCR kit (Qiagen, Hilden, Germany) on a CFX96 qRT-PCR detection system (BioRad, Hercules, CA, USA) with a forward primer (5′-TTT TCA ATC CTC AAC CGT AAG GC-3′), reverse primer (5′-CAG TCC GGT CCC AGA ATG TG-3′), and TaqMan MGB custom probe (5′-6FAM-CAT GTG CCG CCC CAT CGC TGC-MGBNFQ-3′) (Applied Biosystems, Waltham, MA, USA) [27]. The following thermal cycling conditions were used: reverse transcription at 50 °C for 15 min, DNA polymerase activation at 95 °C, and 45 cycles of 95 °C for 30 s followed by 60 °C for 1 min. Genomic equivalents were calculated based on a curve produced with a target specific synthetic RNA standard. Samples with less than 1000 genome equivalents (GEq) per μL were below the lower limit of quantification (LLOQ) and samples with no detectable amplification were reported as below the LOD.

### 2.5. Figures and Statistics

Schematics presented in figures were made with BioRender (created with BioRender.com). GraphPad Prism version 9 for Windows (GraphPad Software, San Diego, CA, USA) was used to generate all graphs and the statistics as reported in figure legends.

## 3. Results

### 3.1. Favipiravir Treatment of Mouse-Adapted Zaire ebolavirus in BALB/c Mice

BALB/c mice challenged with maEBOV and treated with 300 mg/kg favipiravir survived to the end of the study regardless of dosing route (PO, SC, or IP) or dosing schedule (once daily (QD) or twice daily (BID)) (Figure 1, left panel). One mouse in the group treated PO QD had a clinical score of 3 on days 4–6 corresponding to decreased grooming and orbital tightening which resolved by day 7. Two mice in the PO BID treatment group had a clinical score of 2 on day 9 which resolved the following day (Figure 1, right bottom panel). Weight loss was not detected in treated groups (Figure 1, right middle panel). Mice in the vehicle-only control group developed weight loss and increasing clinical scores starting at day 2 following challenge, and all succumbed to infection between 4 and 7 days following challenge (Figure 1, top right panel).

### 3.2. Favipiravir Treatment of Guinea Pig-Adapted Zaire ebolavirus in Hartley Outbred Guinea Pigs

Guinea pigs challenged with gpaEBOV receiving treatment tended to have increased survival compared to vehicle-treated controls regardless of the route or duration of treatment (Figure 2A), although no group was completely protected against lethal disease. Mantel–Cox log-rank tests were used to determine the significance of survival curves. All the treatment groups had significantly longer survival (undefined for all groups except for 11-day PO treatment which had a mean survival of 13.5 days post challenge) than vehicle control groups (mean survival of 7.0 days post challenge). There was no significant difference in survival between duration of treatment in the PO or IP groups, and there was no significant difference in survival between PO and IP treatments. All animals in the vehicle-only control groups succumbed to infection between day 2 and day 11 post challenge. All control groups displayed weight loss starting on day 3 or 4 post challenge and had increasing clinical scores starting on day 4 post challenge, which progressed until euthanasia. Treated groups tended to have less weight loss compared to controls, with surviving animals having recovered their original weight by 14 days post challenge. Clinical signs were not apparent in treatment groups until 6 days post challenge and resolved in all surviving animals by 17 days post challenge. A slight trend for less weight loss and lower clinical scores in the IP group compared to the PO group was observed. All groups displayed elevated body temperature with similar kinetics during the first 10 days post challenge (Figure 2F,G). Hypothermia was observed in animals showing late-stage disease.

Significantly higher viremia was detected in vehicle-only compared to treated guinea pigs in all groups by both qRT-PCR and plaque assay using serum collected during euthanasia, except for the 11-day PO treatment which had no significant difference in qRT-PCR viremia load. Animals surviving to the end of the study tended to have undetectable viral loads (Figure 2H–K).

## 4. Discussion

Both the Hartley guinea pig and BALB/c mouse models of EVD have the advantage of using immune-competent animals along with the disadvantage of requiring adaptation of the virus. In BALB/c mice, all treatments with favipiravir were effective at preventing EVD disease. This suggests that the mouse model could be useful for examining the pharmacokinetics of favipiravir combined with EBOV viral infection dynamics to better understand the potential future therapeutic uses of this promising compound. The mouse model suggests that a 300 mg/kg/day dose is sufficient to achieve a therapeutic level of reduction in viral replication regardless of route of administration or dosing schedule. In guinea pig models of the Argentine hemorrhagic fever disease, a 300 mg/kg/day dose of favipiravir reduced mortality, although the 50% effective concentration is higher for EBOV than Junín virus [35,36]. However, the pharmacokinetics of favipiravir in NHPs is complex and has a large variability across animals, likely indicating that increased doses compared to mouse model predictions are needed to successfully treat EVD in NHPs [36,37]. Dose-dependent increased survival and mean time-to-death in NHP treatment models of EVD with favipiravir have been observed [17,28]. During the JIKI trial, an unexpected drop in human serum titers of favipiravir during EVD treatment was also observed [37]. While it has been demonstrated that mice have slower clearance of favipiravir due to liver metabolism than humans and nonhuman primates [38], BALB/c tolerance of a 300 mg/kg/day therapeutic dose is a good starting point to determine in vivo minimum inhibitory concentrations. Future work to better understand the serum concentration of favipiravir achieved in the guinea pig model could be useful in informing improved dosing in future studies using higher species.

Oral dosing is a highly desirable trait in EVD therapeutics as it reduces the managed care burden and increases the safety of caretakers who therefore do not need to handle needles to treat infected patients. However, in rodent models of EVD, IP doses are often easier to administer. We did not identify significant differences between PO and IP dosing (and SC dosing in the mouse model), which suggests that the route of administration is less important in rodent models of favipiravir treatment of EVD.

Additionally, no significant trends in the duration of treatment were identified from the guinea pig treatment experiment. This suggests that 8 days of treatment is likely to be as effective as longer favipiravir treatment regimens. No evidence of viral persistence was seen in the serum of guinea pigs surviving EBOV challenge (Figure 2H–K); however, future studies on the analysis of viral load from tissues and deep sequencing of these viral populations could better inform whether these treatment doses and schedules induce viral mutagenesis (potentially resulting in escape mutants). The outcomes in this study are in line with the previously described efficacy of favipiravir summarized in Table 1. Importantly, this study provides a direct comparison between the duration of treatment and route of dosing in both mouse and guinea pig models. This supports further translational development based on a shorter duration of treatment using oral dosing. Finally, this study provides important preclinical data to support the further development of favipiravir as an EVD countermeasure, potentially in a combinatorial treatment with other small molecule inhibitors or antibodies.

## Figures and Tables

**Table 1:** Summary of Previous Favipiravir Studies.

	Dosing	Route	Tx Duration	Animal Model	Key Findings	Ref
maMARV (Angola)	300 mg/kg starting on D1, D2, D3, or D4 post infection. 75 or 150 mg/kg on D2	PO	8 days	BALB/c mice	Complete survival of 300 mg/kg groups treated at D1 and D2, increased survival in other treated groups.	[16]
EBOV (Gabon 2001)	100 mg/kg, 150 mg/kg or 180 mg/kg twice daily starting at D(-2) with a loading dose of 200 mg/kg or 250 mg/kg	IM	14 days	cynomolgus macaques	Animals receiving 150 and 180 mg/kg had decreased mortality and increased mean time-to-death.	[17]
EBOV (E718)	150 mg/kg twice daily (300 mg/kg/day)	PO	14 days	A129 interferon alpha/beta receptor knockout mice	Complete survival of treated mice.	[22]
gaSUDV (1976/Nzara-Boneface)	150 and 300 mg/kg/day starting at D1, D2, D3, D4 or D5	SC	7 days	Hartley Guinea Pigs	Complete survival in animals treated at D1 and D3. Decreased mortality and increased mean time-to-death in other treated groups.	[23]
EBOV (Mayinga 1976—undefined passage history)	30 and 300 mg/kg/day starting at D2, D4, or D6	PO	8 days	A129 interferon alpha/beta receptor knockout mice	Complete survival of all treated groups.	[24]
SUDV (Gulu)	150 mg/kg/day starting at D0 + 1 h	PO	8 days	129S6 STAT-1 knockout	Complete survival of treated mice.	[26]
EBOV (Kikwit)	150 mg/kg/day starting at D0 + 1 h	PO	8 days	C57BL/6J interferon α/β and γ receptor double knockout	Decreased mortality (20% in treated groups vs. 100% in untreated)	[27]
EBOV (Kikwit) or MARV (Angola)	200 mg/kg, 150 mg/kg or 75 mg/kg/day starting at D(-2), D(-1) or D1 with a loading dose of 400 mg/kg, 250 mg/kg, or 75 mg/kg	PO or IV	14 days	cynomolgus macaques	Decreased mortality (83% vs. 100%) in high dose treatment group and increased mean time-to-death following EBOV challenge. Decreased mortality (17% vs. 100%) and increased mean time-to-death following MARV challenge.	[28]

## Data Availability

The original contributions presented in the study are included in the article; further inquiries can be directed to the corresponding author/s.
